# Geometry calibration between X-ray source and detector for tomosynthesis with a portable X-ray system

**DOI:** 10.1007/s11548-017-1557-x

**Published:** 2017-03-25

**Authors:** Kohei Sato, Takashi Ohnishi, Masashi Sekine, Hideaki Haneishi

**Affiliations:** 1 Graduate School of Engineering, Chiba University, Chiba, 263-8522 Japan; 2 Center for Frontier Medical Engineering, Chiba University, Chiba, 263-8522 Japan

**Keywords:** Geometry calibration, Iterative image reconstruction, Tomosynthesis, Portable X-ray system

## Abstract

***Purpose*:**

Tomosynthesis is attracting attention as a low-dose tomography technology compared with X-ray CT. However, conventional tomosynthesis imaging devices are large and stationary. Furthermore, there is a limitation in the working range of the X-ray source during image acquisition. We have previously proposed the use of a portable X-ray device for tomosynthesis that can be used for ward rounds and emergency medicine. The weight of this device can be reduced by using a flat panel detector (FPD), and flexibility is realized by the free placement of the X-ray source and FPD. Tomosynthesis using a portable X-ray device requires calibration of the geometry between the X-ray source and detector at each image acquisition. We propose a method for geometry calibration and demonstrate tomosynthesis image reconstruction by this method.

***Methods*:**

An image processing-based calibration method using an asymmetric and multilayered calibration object (AMCO) is presented. Since the AMCO is always attached to the X-ray source housing for geometry calibration, the additional setting of a calibration object or marker around or on the patients is not required. The AMCO’s multilayer structure improves the calibration accuracy, especially in the out-of-plane direction.

***Results*:**

Two experiments were conducted. The first was performed to evaluate the calibration accuracy using an XY positioning stage and a gonio stage. As a result, an accuracy of approximately 1 mm was achieved both in the in-plane and out-of-plane directions. An angular accuracy of approximately $0.5^{\circ }$ was confirmed. The second experiment was conducted to evaluate the reconstructed image using a foot model phantom. Only the sagittal plane could be clearly observed with the proposed method.

***Conclusion*:**

We proposed a tomosynthesis imaging system using a portable X-ray device. From the experimental results, the proposed method could provide sufficient calibration accuracy and a clear sagittal plane of the reconstructed tomosynthesis image.

## Introduction

In the clinical field, X-ray imaging and X-ray computed tomography (CT) are widely used for visualizing the inside of the human body. Aside from these modalities, tomosynthesis is attracting attention because it can generate tomographic images from several simple X-ray images and thereby realizes a low-dose tomographic imaging technique compared with X-ray CT. However, conventional tomosynthesis devices require a high installation cost because of their mechanical size. Furthermore, there is a limitation in the working range of the X-ray source during image acquisition. We have previously reported a portable X-ray system that can be used for ward rounds owing to the broad working range of the X-ray source [1]. The portable X-ray system consists of an X-ray source and a handheld flat panel detector (FPD). Since the portability is high, it can be easily used in the fields of pediatrics and orthopedics. By using this system, low-cost tomographic imaging may be realized.

Studies on the tomography using a mobile X-ray system have been reported in recent years [2–4]. Park et al. [2, 3] performed a feasibility study for image reconstruction using a circular digital tomosynthesis method based on compressed-sensing theory. These reports mostly assumed that the X-ray source moves along a predetermined straight-line path or circular arc path around the subject during image acquisition. This assumption enables the geometric relationship between the X-ray source and the detector to be easily obtained from the X-ray system. Image reconstruction problems can be formulated if the image acquisition geometry is known. However, if the image acquisition path of the portable X-ray system is undefined, the geometric relationship between the X-ray source and the detector at each image acquisition must be measured for image reconstruction.

Some approaches to identify the 3D positional correspondence from 2D X-ray images have been proposed [4–8]. Rottman et al. [4] used a gravitational acceleration vector measured by a microelectromechanical systems sensor with $9^{\circ }$ of freedom as an initial orientation. Image reconstruction and geometry calibration were alternately conducted until a fine quality image was obtained. The processing time may become large because image reconstruction is generally a time-consuming process. Moura et al. [5] used a scaling object with a laser rangefinder to measure the initial geometric parameters. To achieve an accurate geometry calibration, a bi-planar imaging and an optimization process based on anatomical landmark detection were required. This may only produce a superior performance in a limited number of applications and devices. Even if external devices are introduced in the geometry calibration, additional optimization processes are required and such processes lack versatility. Some approaches using calibration markers have also been reported [6–8]. Cheriet et al. [6] performed a calibration using markers embedded in a jacket worn by the patient during image acquisition. Mitton et al. [7] proposed a calibration method using a stereo system in addition to metallic markers. Schumann et al. [8] described a concept based on simulated projections using a simple calibration object. These methodologies require performing the geometry calibration separately from the image acquisition of the subject at every change of the imaging geometry.

From such a background of studies, we have proposed an image processing-based calibration method using a flat calibration object (CO) attached to the X-ray source housing [1]. The CO has high-attenuation metallic grid lines at its periphery, and its image is always captured together with the target image. By attaching the CO to the X-ray source, it is possible to perform the geometry calibration without external devices. In addition, the simultaneous image capturing of the target and the CO could enable both the geometry calibration and the image reconstruction.

In the proposed method, the CO has metallic grid lines placed symmetrically. Although we have confirmed the effectiveness of this method, a large error was obtained in the out-of-plane direction because the CO did not provide rich depth information. For this reason, the reconstructed image quality was not sufficient. Herein, we propose an asymmetric and multilayered calibration object (AMCO) to improve the accuracy of the calibration. In addition, we conduct tomosynthesis image reconstruction to confirm that the proposed system can provide an acceptable tomographic image. In the following section, details of the calibration method with the AMCO are described. Experiments for the evaluation of the calibration accuracy and tomosynthesis image reconstruction are explained in the “Experiments” section. The “Results and discussion” section presents the results and discussions of the performed experiments.

## Methods

### Asymmetric and multilayered calibration object (AMCO)

**Fig. 1:**
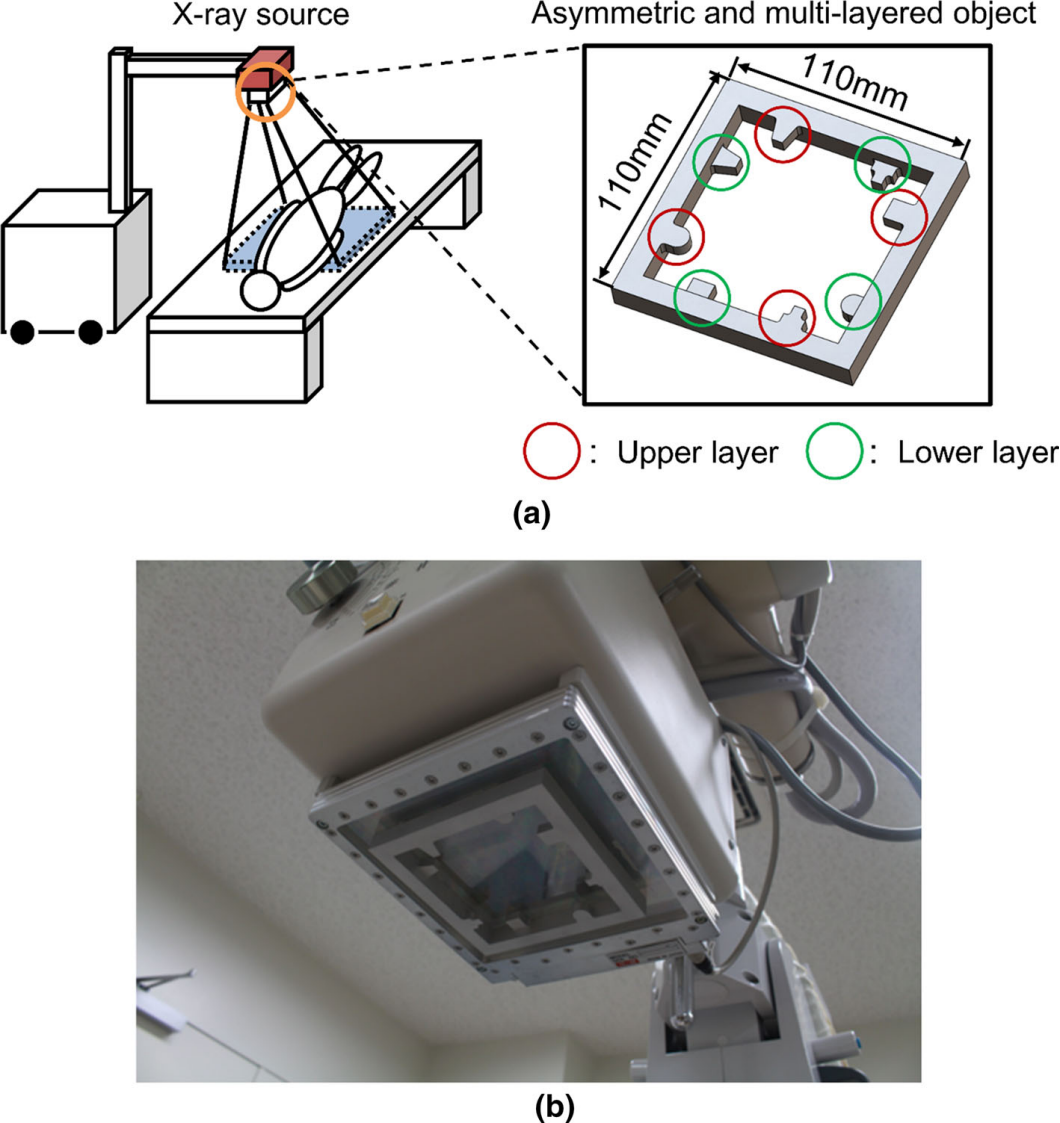
AMCO attached to the X-ray source. **a** Schematic illustration of the imaging system with the AMCO. **b** Photograph of the X-ray source with the AMCO

In our previous study [1], the CO was a single layer with metallic grid lines that were placed symmetrically. If the CO is a single layer, the registration accuracy in the out-of-plane direction becomes significantly lower than that of the in-plane. To avoid this problem, we propose an asymmetric and multilayered calibration object (AMCO). The AMCO is a rigid body object, and its shape is exactly known, as it is generated by computer-aided design-based machining. The vertical difference between layers is reflected in the projection image at different magnifications. Furthermore, the shape and the placement of the CO patterns are designed asymmetrically to avoid the local solution in the optimization. Figure 1a shows a schematic illustration of the portable X-ray system with the AMCO. The AMCO is always attached to the housing of the X-ray source during the target image acquisition, as shown in Fig. 1b, and its projected patterns on the detector are used for calibration of the imaging geometry.

**Fig. 2:**
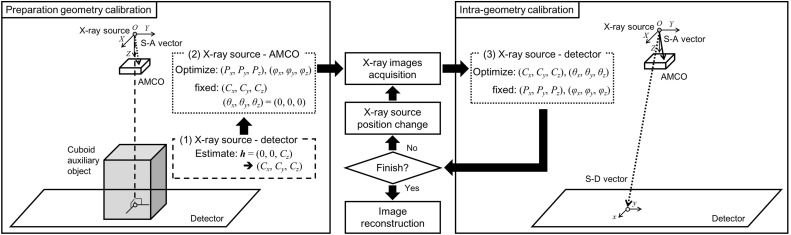
Processing flowchart for tomosynthesis using a portable X-ray system

### Geometry calibration

#### Outline

An outline of the processing for the calibration and image reconstruction using a portable X-ray system is shown in Fig. 2. Before the actual image acquisition, the geometric relationship between the X-ray source and the AMCO must be obtained. During the actual image acquisition, X-ray images are acquired from the free X-ray source positions. The image processing-based calibration is then performed to obtain the geometric relationship between the X-ray source and the detector at each image acquisition. The geometry relationships are estimated by a 2D–3D registration algorithm [9–11], as described in the following section. The image reconstruction is performed using the obtained geometric relationship.

**Fig. 3:**
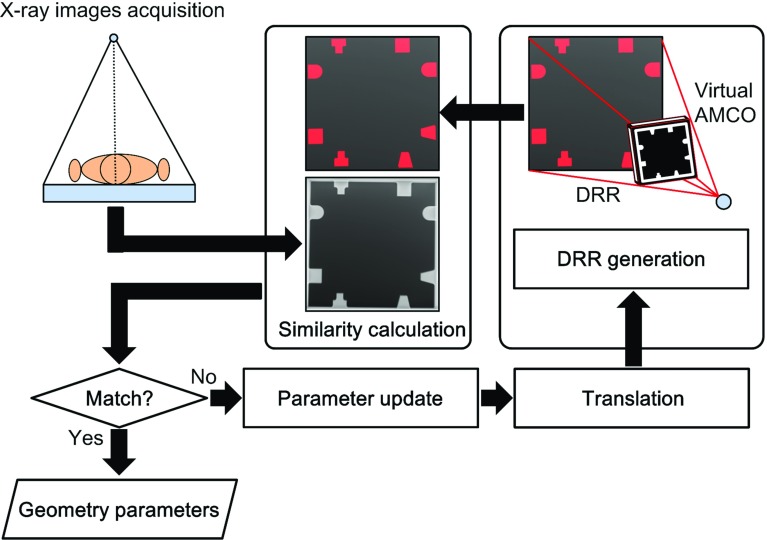
Detailed flow of the geometry calibration

When the AMCO is initially attached to the X-ray source housing, the geometric parameters, namely the position and the tilt of the AMCO relative to the point source of the X-ray, are not necessarily known. Herein, the geometry between the X-ray source and the AMCO is termed as the S–A geometry. The parameters of the S–A geometry are estimated using an image of the AMCO on the detector. In this estimation, the position of the detector relative to the X-ray source is also needed. The geometry between the X-ray source and the detector is termed as the S–D geometry. This estimation can be carried out using a cuboid auxiliary object whose dimensions are known ((1) in Fig. 2). Using the estimated parameters of the S–D geometry, those of the S–A geometry can be obtained ((2) in Fig. 2). This calibration is conducted once before image acquisition. If the AMCO remains attached for daily use, this calibration can only be performed during maintenance. The proposed method, which does not require a calibration object on the patient’s side at each image acquisition, is very simple compared with other procedures.

In the image acquisition for tomosynthesis reconstruction, the parameters of the S–A geometry are used to obtain those of the S–D geometry ((3) in Fig. 2). If the S–D vector can be obtained, it is possible to perform an iterative image reconstruction using the virtual projection.

#### Geometric relationship between the X-ray source and the AMCO

First, the coordinate and parameters are defined, as shown in Fig. 2. We let the point source of the X-ray be the origin, *O* in the world coordinate. We then arbitrarily define a standard point of the AMCO (AMCO-SP). We let the vector from *O* to the AMCO-SP be ${{\varvec{P}}}=\left( {P_x ,P_y ,P_z } \right)$, which we term as the S–A vector. The other parameters of the AMCO are the rotation angles. We define the rotation angle around *x*, *y*, and *z* as ${\varvec{\varphi }} =\left( {\varphi _x ,\varphi _y ,\varphi _z } \right)$, being the origin of the world coordinate as the rotation center. ${{\varvec{P}}}$ and ${\varvec{\varphi }}$ are the parameters of the S–A geometry. In addition, we also define the detector center as ${{\varvec{C}}}=\left( {C_x ,C_y ,C_z } \right)$, which we term as the S–D vector and the rotation angles of the detector to all be zero, ${\varvec{\theta }} =\left( {\theta _x ,\theta _y ,\theta _z } \right) =\left( {0,0,0} \right)$.

**Fig. 4:**
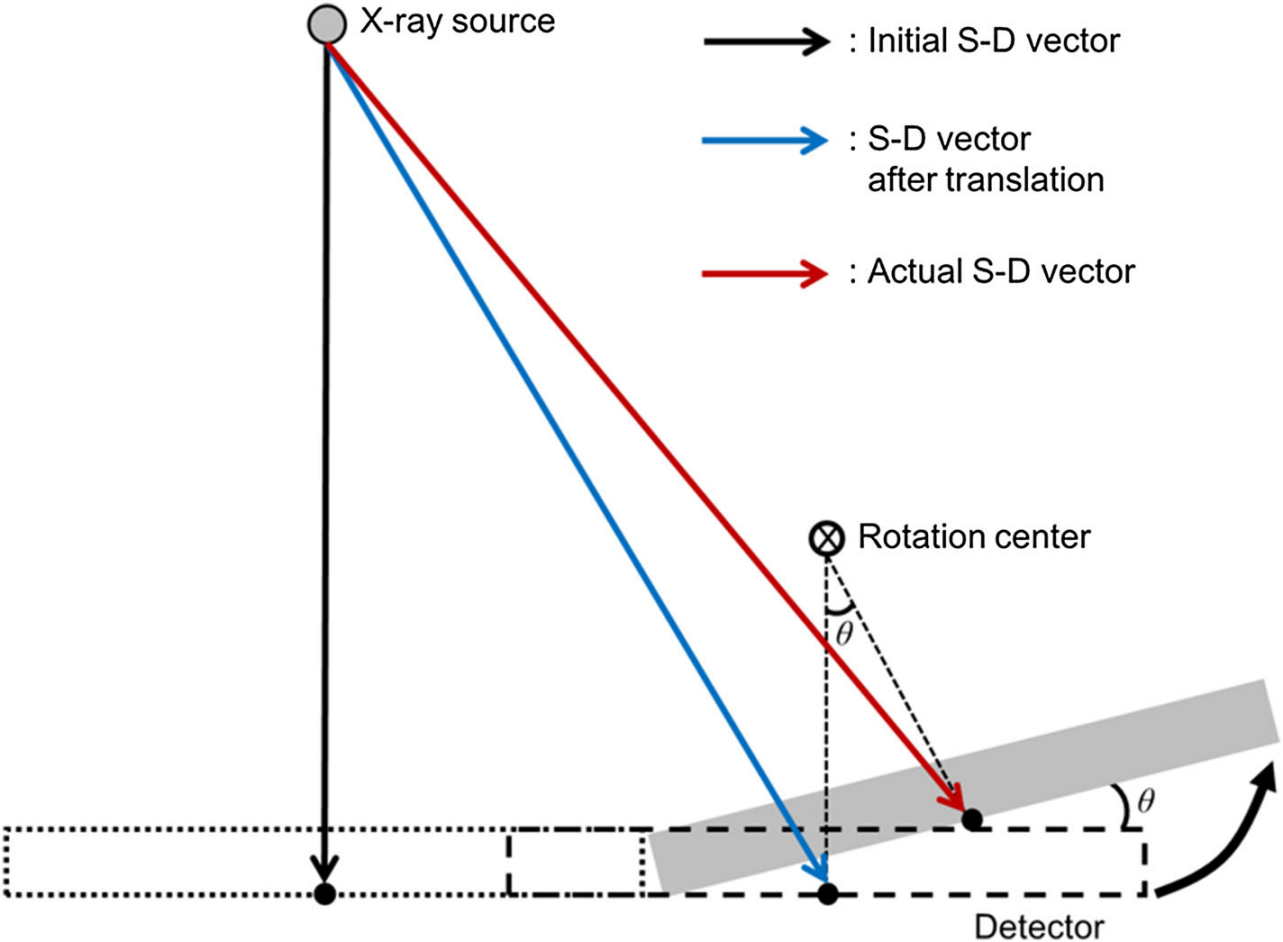
Calculation of an actual S–D vector in the accuracy evaluation experiment of the geometry calibration using an XY positioning stage and a gonio stage

To obtain ${{\varvec{P}}}$ and ${\varvec{\varphi }}$, we use an image of the AMCO on the detector for a certain S–D geometry. The parameters of the S–D geometry are estimated using a cuboid auxiliary object with a metal ball embedded at each corner in a similar way to that reported previously [12]. Next, the parameters of the S–A geometry must be estimated. This estimation is performed by the 2D–3D registration technique, as shown in Fig. 3. Since the 3-D distribution of the linear attenuation coefficient of the AMCO is known, its projection data under an estimated S–D geometry can also be calculated with a computer as a digitally reconstructed radiograph (DRR). Here, the DRR is calculated using the ray-summation method [13]. As we also obtain real projection data, the normalized cross correlation (NCC) [14, 15] between the DRR and real projection data is used as a cost function to optimize the S–A geometry. In this study, after the initial values for these parameters are provided empirically, the Powell-Brent method is used for optimization [16]. It takes a long time to calculate the 2D–3D image registration if the DRR generation is computed using a CPU. Thus, we perform the calculation with a graphics processing unit (GPU) and compute unified device architecture (CUDA) [17, 18].

#### Geometric relationship between the X-ray source and the detector

In the image acquisition of a target subject for tomosynthesis reconstruction, the parameters of the S–D geometry at each image acquisition must be estimated. The way to achieve this is very similar to the above-mentioned process. The NCC between the DRR and real projection data is evaluated, and the optimal parameters of the S–D geometry are found so as to maximize the NCC. In practice, the target subject is also imaged with the AMCO in this step. The overlap of the projected images of the subject and the AMCO affects the parameter estimation. This issue is discussed in the following section.

### Experiments

We evaluated the proposed method in two ways. The first method was a quantitative evaluation of the geometry calibration using an XY positioning stage and a gonio stage. The second method was a visual assessment of the reconstructed tomosynthesis image.

### Experimental devices

We used a portable X-ray system (Canon Inc., Japan) and an FPD (CXDI-50RF, Canon Inc., Japan) for image acquisition. The tube voltage and the tube current were set to 80 kV and 0.80 mAs, respectively. The size of the X-ray image was 2208 $\times$ 2688 pixels, and each pixel had 16 bits. The pixel size was 0.16 $\times$ 0.16 mm$^{2}$. In this experiment, the AMCO was made of aluminum alloy (A5052), its horizontal size was 110 $\times$ 110 mm$^{2}$, and the thickness was 12 mm.

### Geometry calibration

The FPD was placed on an XY positioning stage whose position could be translated in the in-plane direction with a 0.01-mm pitch and a gonio stage that could be tilted with a 1.0-degree pitch. The rotation center of the gonio stage was at a height of 100 mm from the stage surface. The X-ray source was fixed, and the X-ray source-detector distance (SDD) was approximately 900 mm. Two hundred and forty-five X-ray images were acquired by moving the stages in the in-plane direction and tilting around the *x*-axis. The movement interval was 5 mm in the *x*- and *y*-directions, and the tilt interval was $3^{\circ }$.

We evaluated the calibration accuracy in the following way. At the initial setup, the S–D vector, ${{\varvec{C}}}=\left( {C_x , C_y ,C_z } \right)$, was accurately estimated by the method described above. Here, the S–D vector obtained for this setup was defined as the initial S–D vector ${{\varvec{C}}}_{\mathbf{0}} =\left( {C_{x0} , C_{y0} ,C_{z0} } \right)$. For each position of the stage, the S–D vector ${{\varvec{C}}}$ and the rotation vector ${\varvec{\theta }}=\left( {\theta _x ,\theta _y ,\theta _z } \right)$ were estimated by the proposed calibration method. Then, the difference between the components of the actual S–D vector and those of the estimated S–D vector was evaluated. In addition, the difference between the components of the rotation vector and those of the estimated rotation vector was evaluated. Each component of the actual S–D vector ${{\varvec{C}}}$ and the rotation vector ${\varvec{\theta }}$ is represented as:

1 $$\begin{aligned} C_x^{lmn}= & {} C_{x0} +5l,l=-3,-2,-1,0,1,2,3 \nonumber \\ C_y^{lmn}= & {} C_{y0} +5m+100\times \sin \theta _x^{lmn} ,m=-3,-2,-1,0,1,2,3 \nonumber \\ C_z^{lmn}= & {} C_{z0} -100(1-\cos \theta _x^{lmn} ) \nonumber \\ \theta _x^{lmn}= & {} 3n,n=-2,-1,0,1,2 \nonumber \\ \theta _y^{lmn}= & {} \theta _z^{lmn} =0 \end{aligned}$$

Namely, the actual vector was given from a known tilt angle of the gonio stage as ${\varvec{\theta }} ^{lmn}=\left( {\theta _x^{lmn} ,0,0} \right)$, and the actual S–D vector was calculated from the initial S–D vector and a known moving distance of the XY positioning stage and the tilt angle, as shown in Fig. 4. The estimated vectors are represented as ${\widehat{{{\varvec{C}}}}}^{lmn}=\left( {{\widehat{C}}_x^{lmn} ,{\widehat{C}}_y^{lmn} , {\widehat{C}}_z^{lmn} } \right)$ and ${\widehat{{\varvec{\theta }}}}^{lmn}=\left( {{\widehat{\theta }} _x^{lmn} ,{\widehat{\theta }} _y^{lmn} , {\widehat{\theta }}_z^{lmn} } \right)$. The error was then evaluated as:

2 $$\begin{aligned} e_{in}^{lmn}= & {} \sqrt{(C_x^{lmn} -\widehat{{C}}_x^{lmn} )^{2}+(C_y^{lmn} -\widehat{{C}}_y^{lmn} )^{2}} \nonumber \\ e_{out}^{lmn}= & {} \left| {C_z^{lmn} -\widehat{{C}}_z^{lmn} } \right| \\ e_{tilt}^{lmn}= & {} \left| {\theta _x^{lmn} -\widehat{{\theta }}_x^{lmn} } \right| \nonumber \end{aligned}$$

**Fig. 5:**
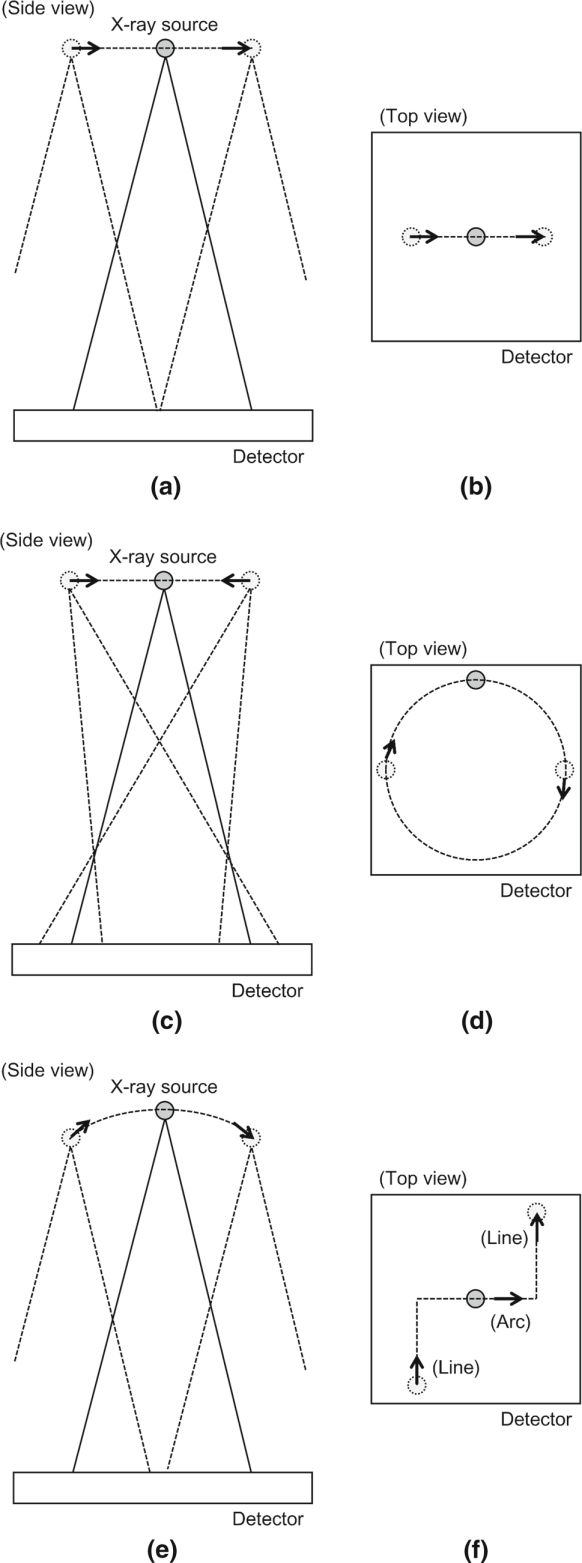
*Side view* and *top view* of the acquisition trajectories. **a, b** Simple line. **c, d** Circular. **e, f** Complex

### Tomosynthesis image reconstruction

The algebraic reconstruction technique (ART) [19–23], which is one of the simple and widely used iterative reconstruction techniques, was used for image reconstruction. In addition, total variation (TV) [23–25] which is an effective sparsifying transform for medical images, was also applied. TV minimizing significantly preserves edges and creates a smoother image. The use of this method has a potential to reduce the influence of the missing data owing to the small number of image acquisitions. An image is estimated by minimizing the following:

3 $$\begin{aligned} {\widehat{\mathbf{f}}}=\mathop {\min }\limits _\mathbf{f} [\left\| {C\mathbf{f}-\mathbf{y}} \right\| _2^2 +\lambda \left\| {\hbox {TV}(\mathbf{f})} \right\| _1 ] \end{aligned}$$

where **f** represents the reconstructed image, **y** represents the acquired data, and *C* is the coefficient matrix of the detection probability. $\lambda$ is the smoothing parameter that controls the trade-off between the data fidelity and TV term. $\left\| \right\| _2$ and $\left\| \right\| _1$ represent the $\hbox {L}_{2}$ norm and the $\hbox {L}_{1}$ norm of a vector, respectively. The gradient descent method [16, 26] was used to minimize Eq. 3. In this paper, $\lambda$ was empirically set to 0.5 by visual inspection.

**Table 1:** Mean and maximum error of the geometry estimation

Tilt angle of the gonio stage	AMCO		CO	
	Mean	Maximum	Mean	Maximum
$$-6^{\circ }$$				
In-plane (mm)	1.89	3.58	0.79	1.57
Out-of-plane (mm)	0.61	1.59	3.26	6.05
Tilt angle $({^\circ })$	0.33	1.39	1.70	6.44
$$-3^{\circ }$$				
In-plane (mm)	0.69	1.40	0.44	0.87
Out-of-plane (mm)	0.34	1.11	2.28	4.12
Tilt angle $({^\circ })$	0.40	1.54	3.65	4.93
$$0^{\circ }$$				
In-plane (mm)	0.63	1.23	0.46	1.01
Out-of-plane (mm)	0.66	2.54	1.55	2.81
Tilt angle $6({^\circ })$	0.83	1.87	0.72	1.21
$$3^{\circ }$$				
In-plane (mm)	1.40	3.35	0.67	1.19
Out-of-plane (mm)	0.41	1.46	0.98	2.14
Tilt angle $({^\circ })$	0.59	1.64	1.92	3.00
$$6^{\circ }$$				
In-plane (mm)	2.70	5.82	0.93	1.70
Out-of-plane (mm)	0.96	2.70	2.23	5.18
Tilt angle $({^\circ })$	0.71	1.40	1.49	4.93

Three image acquisition tests were conducted to confirm the robustness by the trajectory of the X-ray images acquisition. A foot model phantom was used as the subject to be reconstructed. Twenty-one X-ray images were obtained in each image acquisition. The acquisition trajectories were simulated as simple line, circular, and complex trajectories as shown in Fig. 5. For the simple line trajectory, the FPD was translated from −50 to 50 mm with 5-mm intervals. For the circular trajectory, the FPD was translated along the circle in the *xy*-plane with a radius of 50 mm at regular intervals. The complex trajectory was a combination of the line and arc trajectories. In the line trajectory part, the FPD was translated from −50 to 50 mm with 10-mm intervals. In the arc trajectory part, the FPD was tilted from $-10^{\circ }$ to $10^{\circ }$ with 2-degree intervals and the translation of the FPD was 0 mm. To achieve the above trajectories, the translation and tilt were controlled by an XY positioning stage and a gonio stage. The X-ray source was fixed, and the SDD was approximately 900 mm, similar to that in the previous section. Before image reconstruction, the patterns of the AMCO were manually eliminated from the projection image.

**Fig. 6:**
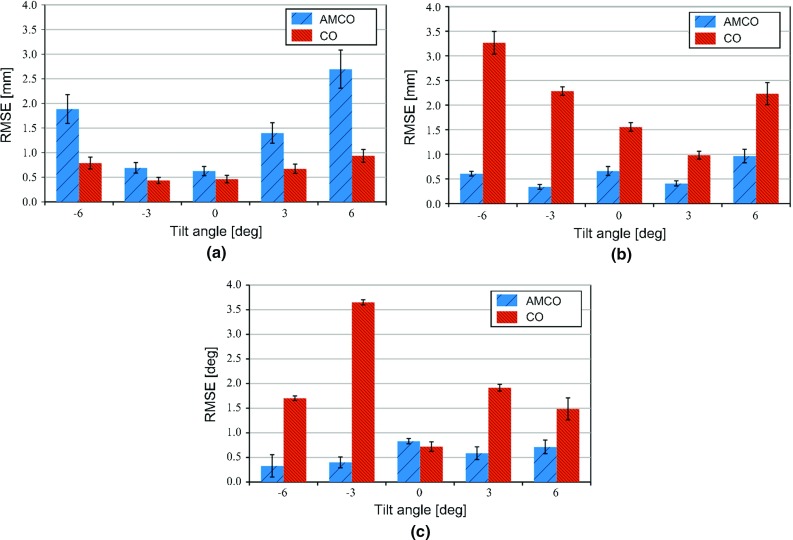
Comparison of the root-mean-square errors at each angle of the gonio stage. Each *error bar* represents the standard error. **a** In-plane direction. **b** Out-of-plane direction. **c** Tilt direction

## Results and discussion

### Geometry calibration

**Fig. 7:**
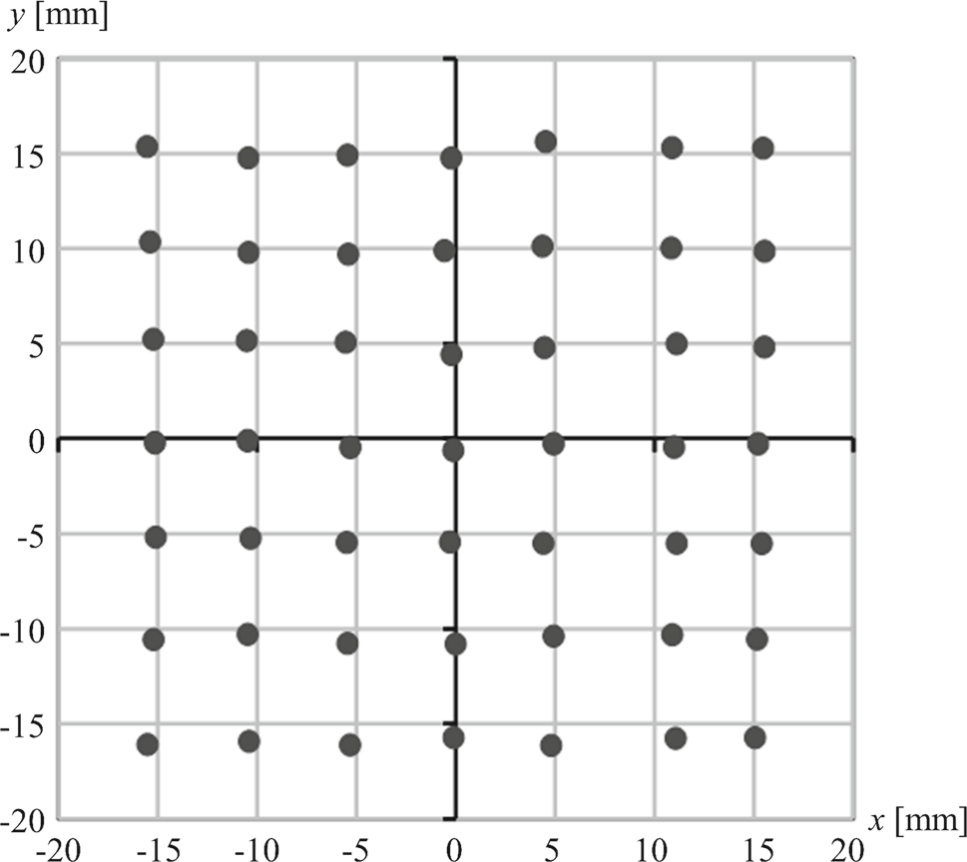
*Plots* of the estimated in-plane S–D vectors. Each black dot represents the estimated S–D vector. The ideal S–D vector is the nearest grid point

Table 1 and Fig. 6 show the calibration error of the geometry estimation defined by Eq. 2, and Fig. 7 shows the plots of the estimated in-plane S–D vectors $\left( {\theta _x =0} \right)$. The results using a CO obtained from our previous report [1] are also indicated in Table 1 and Fig. 6. The origin in Fig. 7 represents the position at the initial step. The mean errors of the 245 images in the in-plane direction and the out-of-plane direction with the previously used CO were 0.66 mm and 2.06 mm, whereas those with the AMCO were 1.46 mm and 0.60 mm, respectively. Furthermore, the mean error of the tilt angle with the CO was $1.90^{\circ }$ and with the AMCO was $0.57^{\circ }$. Although only the results in the in-plane direction with the AMCO had a large variance compared with the CO, we confirmed that stable geometry estimations could be performed without large outliers. Cheriet et al. [6], who used markers embedded in a jacket, reported a mean error of 0.97 mm and $0.3^{\circ }$. Schumann et al. [8], who placed a calibration object on the patient’s side, reported a calibration accuracy of 1.41 mm, $0.62^{\circ }$ (the anteversion angle) and $0.89^{\circ }$ (the inclination angle). We achieved an accuracy comparable with these reports without the requirement of external devices or the placement of a calibration object for each image acquisition.

By using the previous CO, the error in the out-of-plane direction was large because the CO did not provide rich depth information. By doubling the layers, the calibration accuracy was improved. The error in the in-plane direction with the AMCO was slightly larger than that obtained with the CO. However, we noticed that with the CO in certain initial positions, improper convergences were observed because of the symmetric pattern property of the CO. Conversely, the registration using the AMCO always exhibited stable convergences. Therefore, combining an AMCO and an image processing-based calibration method would be a useful approach.

**Fig. 8:**
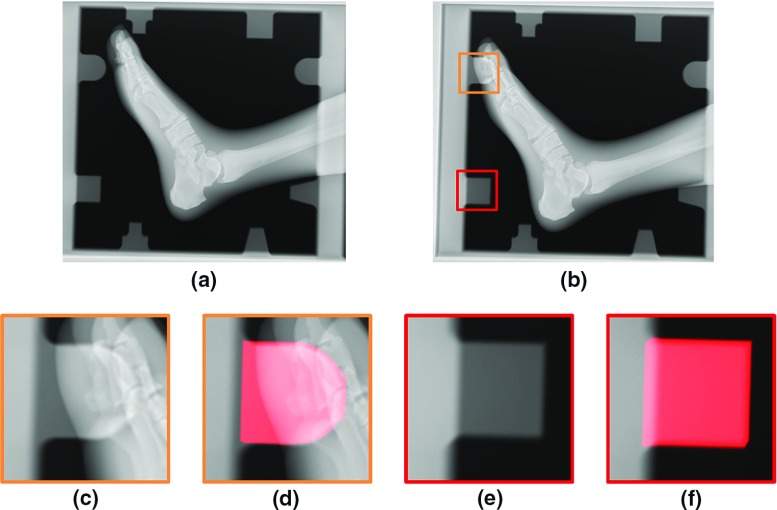
Acquired X-ray images. **a** An example where the error in the in-plane direction was small (*x* = −20 mm, *y* = 0 mm). **b** An example where the error in the in-plane direction was comparatively large (*x* = 15 mm, *y* = −20 mm). **c, d** Enlarged image and the superposition with DRR (*red*) around the *yellow square* in (**b**). **e, f** Enlarged image and the superposition with DRR (*red*) around the *red square* in (**b**)

However, it was confirmed that S–D vectors around $x = 10$ mm generally resulted in large errors, whereas those around the origin exhibited small errors, as shown in Fig. 7. Figure 8a shows an example of an X-ray image with a small calibration error, and Fig. 8b shows an example of a large calibration error. There were some areas which overlapped with the AMCO and the foot model phantom, as shown in Fig. 8c, d. In principle, the DRR is only generated from the volume data of the AMCO, while the real projection data include the subject in addition to the AMCO. The current similarity measure might easily be affected by such a difference. Therefore, an accurate geometry calibration may be performed only when all AMCO patterns can be projected clearly, as shown in Fig. 8e, f. To improve the registration accuracy, a robust similarity measure of the overlap with the subject should be introduced.

In addition to the similarity measure mentioned above, the design of the AMCO is also important. The image contrast depends on the material used for the AMCO. Because the calibration method is based on image processing using the image contrast of the projected patterns, the material of the AMCO is an important factor. The accuracy may be further improved by optimizing the pattern and material of the AMCO. Herein, the shape and placement of the patterns and the material were determined by the ease of processing. Therefore, determination of the optimal parameters of the AMCO design for the proposed tomosynthesis system should be performed.

In terms of the computational time, it took approximately 5 seconds per image to complete an S–D geometry calibration. A greater efficiency may be possible by a downsampling of the used images in the optimization.

**Fig. 9:**
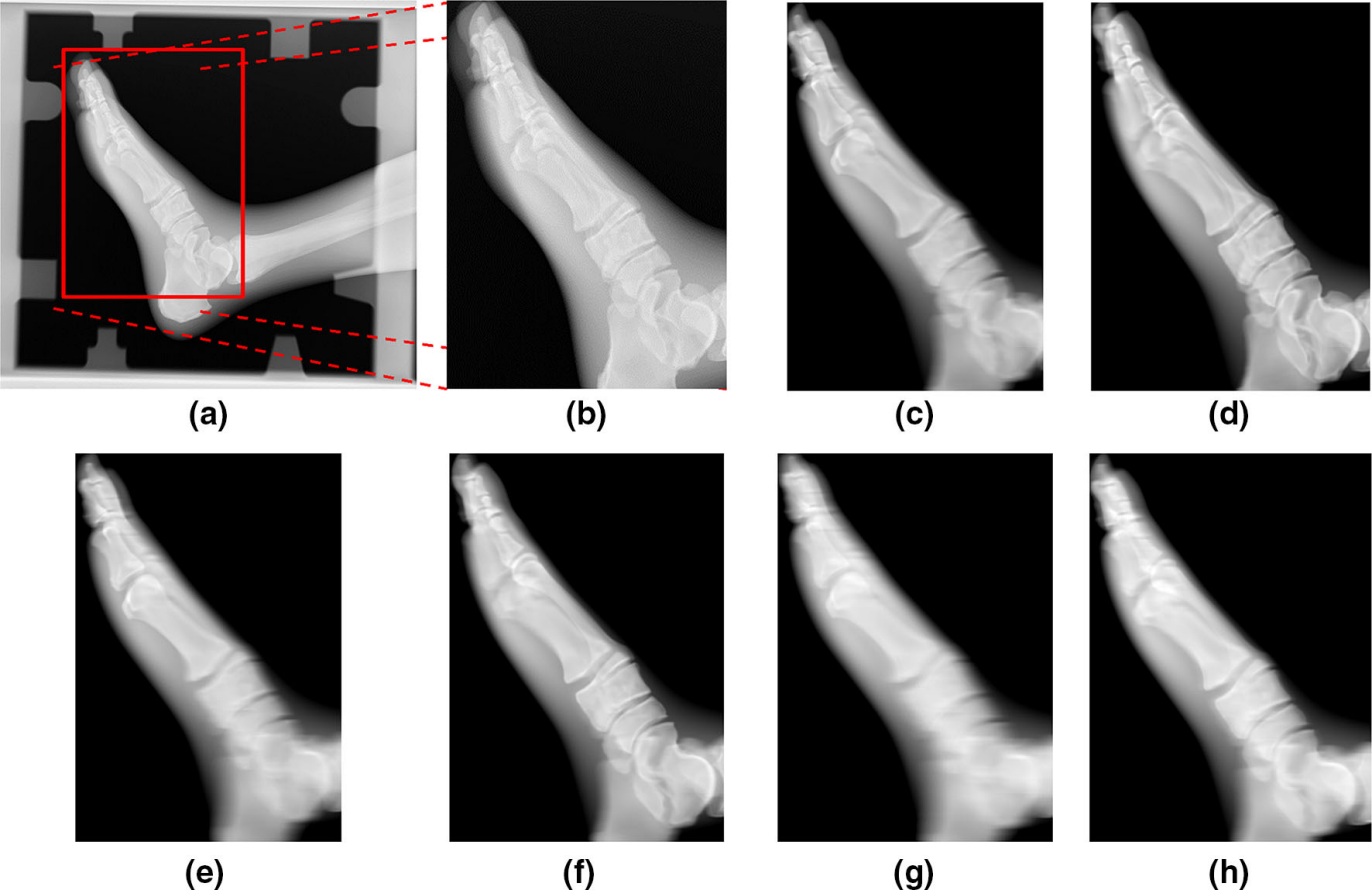
X-ray image and reconstructed tomosynthesis. **a** Original X-ray image. **b** Enlarged image around the *red rectangle* in (**a**). **c, d** Tomosynthesis images obtained by changing the depth of interest (line trajectory). **e, f** Tomosynthesis images by changing the depth of interest (circular trajectory). **g, h** Tomosynthesis images by changing the depth of interest (complex trajectory)

**Fig. 10:**
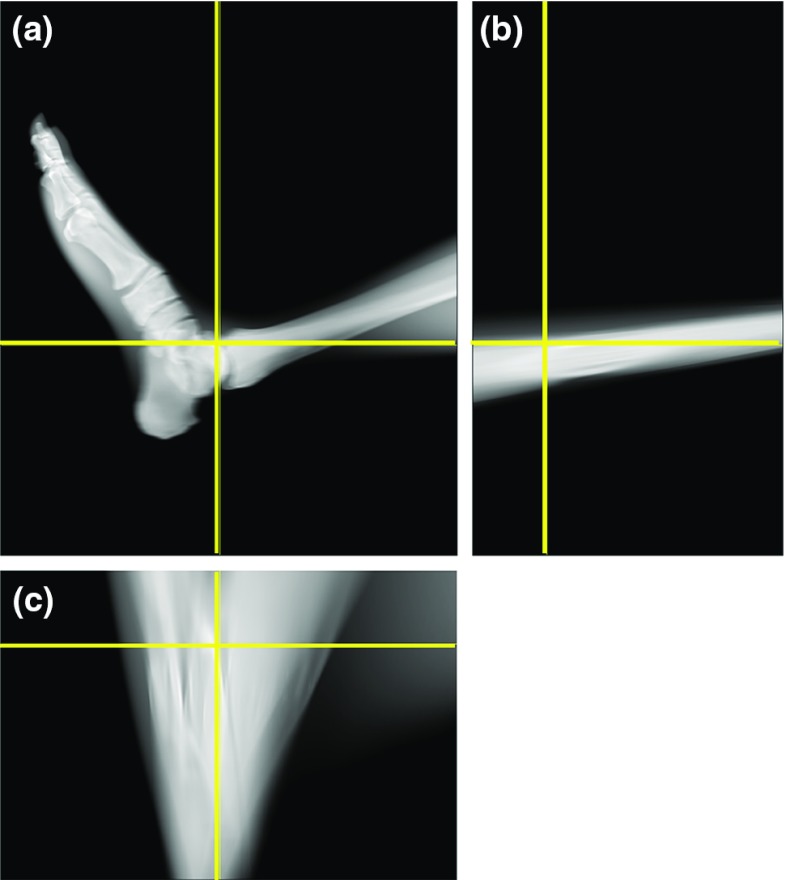
Cross-sectional images of the reconstructed tomosynthesis images. **a** Sagittal plane. **b** Coronal plane. **c** Axial plane

### Tomosynthesis image reconstruction

Figure 9a, b shows a simple X-ray image, and Fig. 9c, d shows the reconstructed images acquired from the line trajectory which were focused at different depths. In a similar way, Fig. 9e–h shows the reconstructed tomosynthesis images acquired from the circular and complex trajectories under the same depth condition as Fig. 9c, d. The bones at multiple depths were overlapped in the simple X-ray image, and it was difficult to identify each bone. However, the reconstructed images could clearly show the bone and joint space at an arbitrary depth. From a comparison between Fig. 9c, d, we could confirm that the tomographic planes at different depths could be observed from the reconstructed tomosynthesis image. Furthermore, in the second and third reconstruction cases, it was possible to obtain similar tomographic images, as shown in Fig. 9e–h, even if images acquired under the circular and complex trajectories were used for image reconstruction. Whereas some differences were observed between reconstructed images obtained from different acquisition trajectories, we confirmed that all reconstructed images exhibited an enhanced bone structure at different depths. In addition, Fig. 10 shows the cross-sectional images of the reconstructeds 3-D image in Fig. 9c. As shown in Fig. 10a, only the sagittal plane could be clearly imaged, but from the unclear images (Fig. 10b, c), we confirmed that the tomosynthesis exhibited an incomplete tomography because of the missing data.

In this paper, we showed the reconstruction from 20 images. However, the relationship between the trajectory, the number or combination of X-ray source positions and the quality of the reconstruction image is a very interesting issue. This issue will be studied in the near future. The image reconstruction algorithm for the proposed system is another interesting topic. In this paper, ART + TV was used in the image reconstruction. In recent reports, a compressed-sensing approach, which reconstructs signals using incomplete measurement data, has been studied, but has not yet been applied to clinical practice [27, 28].

## Conclusion

We proposed a tomosynthesis imaging system using a portable X-ray device. Our system is able to perform image processing-based calibration using an AMCO attached to the X-ray source during target image acquisition. From experimental results, the proposed method was shown to provide a sufficient calibration accuracy of approximately 1 mm and $0.5^{\circ }$. Regarding image reconstruction, only the sagittal plane could be clearly imaged.

Further improvement of the AMCO design and development of the image reconstruction algorithm for tomosynthesis using a portable X-ray system will be undertaken in future work.
